# The Association of Urbanicity with Cognitive Development at Five Years of Age in Preterm Children

**DOI:** 10.1371/journal.pone.0131749

**Published:** 2015-07-10

**Authors:** Marion Gouin, Cyril Flamant, Géraldine Gascoin, Valérie Rouger, Agnès Florin, Philippe Guimard, Jean-Christophe Rozé, Matthieu Hanf

**Affiliations:** 1 Department of Neonatal Medicine, Nantes University Hospital, Nantes, France; 2 National Institute of Health and Medical Research CIC 1413, Nantes University Hospital, Nantes, France; 3 Department of Neonatal Medicine, Angers University Hospital, Angers, France; 4 Réseau “Grandir ensemble”, Nantes University Hospital, Nantes, France; 5 Faculty of psychology, University of Nantes, Research Center of Education, CREN EA 2661, Nantes, France; Institute for Health & the Environment, UNITED STATES

## Abstract

**Objective:**

To determine the association of urbanicity, defined as living in an urban area, with cognitive development at five years of age in preterm children who were free of any disabilities or neurodevelopmental delays.

**Design:**

Prospective population-based cohort.

**Setting:**

French regional Loire Infant Follow-up Team (LIFT) network.

**Participants:**

Included in the study were 1738 surviving infants born between March 2003 and December 2008 before 35 weeks of gestational age. At two years of age, the children were free of any disabilities and neurodevelopmental delays and were living in the Pays de la Loire region from their birth to five years of age.

**Main Outcome Measures:**

The cognitive development at five years of age was evaluated with the Global School Adaptation score (GSA). The urbanicity of the residence for each child was classified into three groups: urban, quasi-rural, and rural area.

**Results:**

Quantile regression approaches were used to identify a significant association between urbanicity and the GSA score at five years of age (adjusting for child and family characteristics). We found that the negative impact of urbanicity on the GSA score was more important for the lower quantile of the GSA scores.

**Conclusions:**

Urbanicity was significantly associated with cognitive neurodevelopment at five years of age in preterm children born before 35 weeks of gestation. Complementary results additionally suggest that this relation could be mediated at the residence level by a high socioeconomic deprivation level. If these results are confirmed, more personalized follow-ups could be developed for preterm children. Further studies are needed to finely identify the contextual characteristics of urbanicity that underlie this association.

## Introduction

Preterm infants are at major risk for adverse neurodevelopmental outcomes [1–7]. From an early stage, they should be carefully monitored to detect any potential adverse consequences and minimize future sequelae through adapted care and interventions [4]. An assessment at the age of two years is critical to diagnose any severe disabilities or sensory deficits [8,9] but is ultimately insufficient in the long run [10]. A supplementary assessment at five years of age is recommended to identify the more subtle problems [11]. These problems include cognitive disorders, learning disabilities, specific neuropsychological deficits in executive function, behavioral problems, and educational underachievement [11], all of which can lead to difficulties in school. Unfortunately, because of the cost and time, this assessment cannot be routinely performed on all preterm children, and it is often restricted to children with a major neurological impairment or born very preterm. Therefore, specific attention is required for preterm children free of any disabilities at age two to determine the true prevalence and nature of neurodevelopmental problems that arise from their premature birth [11].

The factors that affect the cognitive prognosis at five years of age are not well known in this population because the appropriate longitudinal studies assessing neurodevelopment at various ages are scarce and subject to referral bias [12,13]. Nevertheless, previous studies in this area have shown that the cognitive development and school abilities in preterm children free of any disabilities or neurodevelopmental delays at age two are associated with several factors, such as the gestational age, birth weight, and socioeconomic characteristics of the family [12–15]. Although a growing number of studies have documented the significant effects of the contextual environment on the health and outcomes of children [16–19], to the best of our knowledge, there are currently no studies that have identified the particular contextual factors that impact the cognitive development of this particularly vulnerable population.

It has already been shown that contextual factors that affect health are highly interrelated with a child’s residence and its associated level of urbanicity [20]. Studies have found that the rapid urbanization of the world’s population over the past few decades, and the consequent increase in the concentration of urban and suburban populations, has created a characteristic termed urbanicity, defined as living in urban areas, that can have strong adverse or beneficial effects on the health and behavior of children [17,20,21]. As demonstrated by Vlahov and Galea [20], the most common topics of concern that emerge from the urban health literature can be summarized in three principal themes: features of the social environment, features of the physical environment, and the provision of health and social services.

Because of the vulnerability of preterm children who are free of any disabilities or neurodevelopmental delays at age two, we hypothesized that the level of urbanicity of their residence and the associated contextual factors might strongly influence their cognitive development at five years of age, independent of their individual and familial characteristics. If verified, this hypothesis could lead to the development of better adapted preventive measures and more personalized follow-ups for preterm children.

The purpose of this study was to examine: 1) the association of urbanicity with cognitive development of preterm children at five years of age who were free of any disabilities or neurodevelopmental delays at age two and 2) the specific mechanisms through which the contextual characteristics of urbanicity might affect their cognitive development. Data from a French prospective population-based cohort (LIFT study) of preterm children were analyzed with a multilevel method of estimation using quantile regression [22].

## Materials and Methods

### Ethics statement

The LIFT cohort (Grandir ensemble en Pays de la Loire) is registered at the French data protection authority in clinical research (Commission Nationale de l’Informatique et des Libertés or CNIL, No. 851117). The study received the favorable opinion of an ethics committee (GNEDS, Groupe nantais d’éthique en santé). Written consent was obtained from the parents of each child before inclusion.

### Study area and population

This study included all surviving infants born between March 2003 and December 2008 before 35 weeks of gestational age with an optimal neurodevelopmental outcome at two years of age. It was restricted to children that lived in the Pays de la Loire (PDL) region from birth to their five year cognitive evaluation. PDL is one of the 27 administrative regions of France, and it is located on the western Atlantic coast of France. In 2011, the 83,534 km^2^ region had a population of approximately 3,600,000 habitants, concentrated in seven major cities of more than 100,000 habitants each.

The participants were enrolled in the regional Loire Infant Follow-up Team (LIFT) network, which was implemented in 2003 to follow infants born in the PDL at 35 weeks or less of gestational age. The LIFT network includes 24 maternity clinics, of which three have neonatal intensive care units (Nantes, Angers, and Le Mans). The goal of the LIFT network is to screen for early clinical anomalies associated with preterm births and propose adapted care for these instances. Trained physicians monitor the LIFT cohort children in a standardized manner at 3, 9, 12, 18, and 24 months and 3, 5, and 7 years. All children free of any disabilities or neurodevelopmental delays at age two were included in the study. Children with cerebral palsy, blindness, or that required a hearing aid and children with a development delay, defined as a developmental quotient of less than 85 in the absence of a disability or an ages and stages questionnaire score of less than 185 in the absence of developmental quotient were regarded as having an overall “non-optimal neurodevelopmental outcome” at two years of age and were excluded.

### Data collection

Perinatal data (gender, birthweight Z-score, gestational age, and twin status) and socioeconomic information (social benefits for low financial income and socioeconomic level) were collected. The socioeconomic level took into account the parent with the better job according to a scale from lower socioeconomic level to higher socioeconomic level based on an official classification developed by the National Institute for Statistics and Economic Studies (INSEE). The sociodemographic level and social health insurance for low incomes were treated as two-level categorical variables.

### Neurodevelopmental assessment at five years of age: the Global School Adaptation (GSA) score

The GSA score was used to assess the level of neurodevelopment of the preterm children. It was originally designed as a tool for teachers to assess a child’s abilities and behavior in the classroom [23,24]. This score is periodically used by the French ministry of education to follow child’s abilities and behavior in French public schools. In a previous study, our team showed that the validity of this simple questionnaire as a screening tool for adverse neurodevelopmental outcomes in preterm children is very good when compared to the gold standard (IQ score) [25].

At the age of five years ± two months, the questionnaire was given to the parents of the children followed by the network, who then passed it on to teachers. Six questions investigated linguistic competence (school conversation, participation, pertinence, vocabulary, syntax and pronunciation, and understanding), and five questions investigated non-verbal abilities (memory, arithmetic, logical capacities, manual ability, and gross motor coordination). Eight questions addressed the child’s behavior in the classroom (respect of classroom rules, attention, independence when confronted with a task, speed of task execution, work organization, self-confidence, ability to keep up with classroom rhythm, and tiredness). The final question invited the teacher to give their prognosis for the child’s future adaptation to school life. Each question was given a score 1–3, with a higher mark representing a better result. The total score was calculated by adding the points from the 20 questions, resulting in a range of 20–60.

### Assessment of urbanicity

The residential communes (the smallest administrative spatial unit in France) of the children were determined from their addresses collected at their five-year cognitive assessment. The urban unit concept developed by the INSEE was used to define the degree of urbanicity of the communes [26]. An urban unit is a group of communes where no residence is separated from the next by more than 200 meters. Three urban unit categories were used: rural (less than 2000 people), quasi-rural (2000–9999 people), and urban (10,000–1,999,999 people).

### Assessment of the contextual factors associated with urbanicity

To evaluate the potential impact of geographical disparities on the children’s cognitive development, two contextual dimensions were studied at the area level: the neighborhood social environment and the accessibility to child healthcare.

We used a socioeconomic deprivation index, following a previously published method [27], to assess the neighborhood social environment. Four socioeconomic and demographic variables that reflected key dimensions of deprivation were extracted from the 2008 INSEE for all of the communes in the PDL region [27,28]: median household income, percentage of high school graduates in the population aged ≥ 15 years, percentage of persons from lower socioeconomic levels in the active population, and unemployment rate. The first two variables constitute negative dimensions of deprivation, and the last two constitute positive dimensions. After imputation of missing data, a principal component analysis was conducted. This index was defined as the linear combination of the variables on the first axis of the analysis and was then divided according to the 25^th^ and 75^th^ percentiles: the first category comprised the most deprived communes, and the third category comprised the wealthiest ones.

To assess the accessibility of child healthcare, two indicators were used: accessibility to general practitioners (GP) and accessibility to public maternal and child health (MCH) clinics. The accessibility to GP was measured through the IRDES (French Institute for research and information in health economics) measure computed using a “two-step floating catchment area” method [29]. This index was divided according to the 25^th^ and 75^th^ percentiles: the first category comprised the communes with the worst access to GP, and the third category comprised ones with the best access to GP. The accessibility to public MCH clinics was measured at the commune level using the estimated distance (in km) from the town hall of the commune to the nearest public MCH clinic. This index was divided according to the 25^th^ and 75^th^ percentiles: the first category comprised the communes with the best access to public MCH clinics, and the third category comprised ones with the worst access.

### Statistical analysis

Before the analysis, children free of any disabilities or neurodevelopmental delays at age two and with follow-up data at age ≥ 5, with or without missing GSA scores at age five, were compared. Similarly, children with GSA score were compared according to the urbanicity level of their place of residence.

Quantile regression approaches [22], which model conditional quantiles of the response variable, were used. We used a quantile regression which, unlike conventional regression approaches, not only considers the mean tendency of GSA score level regarding covariate changes, but also determines the covariate effect conditional on specific quantiles of GSA score. This method was chosen to better understand whether the determinants of the cognitive development level change at different levels of the GSA score distribution. Moreover, it has the supplementary advantage to quantify the relative loss of GSA score associated with a particular risk factor. To deal with the two-level nature of the data (individual and residential characteristics), linear quantile mixed models, formalized by Geraci and Bottai, were performed [30].

Quantile regression for the median and the 20^th^ and 80^th^ percentiles was simultaneously performed. The standard errors of the coefficients were estimated by bootstrapping with 1000 replications, as suggested elsewhere [31]. The confidence intervals were also computed. To study the impact of urbanicity on the cognitive development of preterm infants, three quantile regression models were developed: no adjustment (model 1), adjustment for the children characteristics (model 2), and adjustment for the children and parent/family characteristics (model 3).

We also examined the specific mechanisms through which the contextual characteristics of urbanicity might affect the cognitive development of preterm infants, using adjustment variables of the model 3 (without urbanicity level) and: deprivation index (model 4), GP index (model 5), and distance to MCH clinic (model 6). All of the analyses were performed with the statistical software R.

## Results

**Fig 1:**
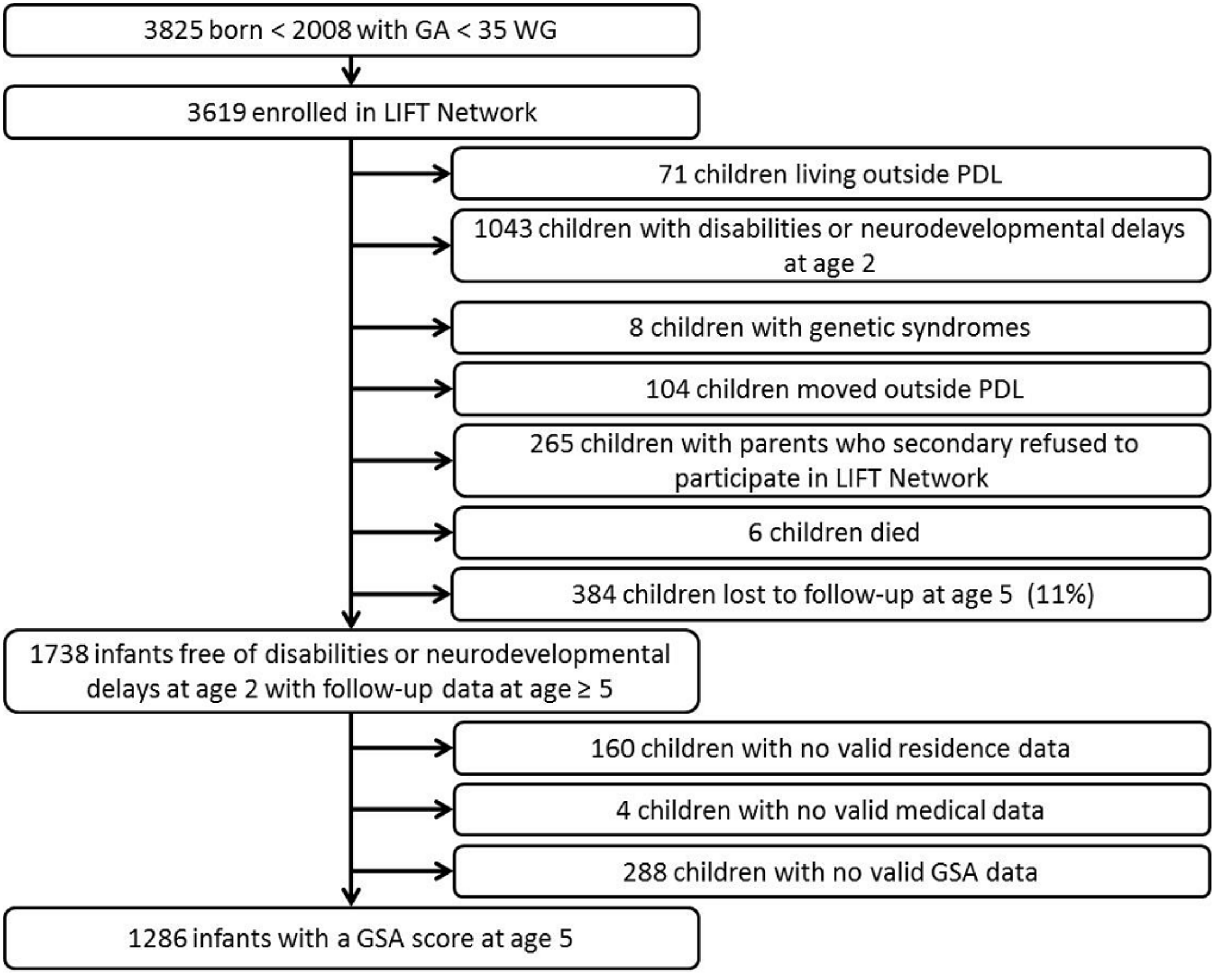
Flow chart.

Between January 2003 and December 2008, 3619 children born before 35 weeks of gestational age were enrolled in the LIFT network. In this study, 71 were excluded because they resided outside our region of interest, 1043 because of a non-optimal neurodevelopmental status at age two, and eight were excluded because of a congenital abnormality. In addition, 104 moved outside our region of interest, 265 had parents who secondary refused to participate in the LIFT cohort, 6 died, and we lost contact with 384 during the five-year follow-up period (11%). Accordingly, the study population only included the 1738 children who underwent a physical examination and psychometric assessment at ages two and five and were considered free of any disabilities or neurodevelopmental delays at age two. Among these children, data on their schooling, residence, perinatal, and sociodemographic characteristics were only available for 1286 (74%) (Fig 1). The characteristics of the study population are summarized in Table 1. The median GSA score was 53 (IQR: 46–57). The sample was composed of 52.5% male and 47.5% female children, the median gestational age was 33 weeks of gestation (WG; interquartile range [IQR]: 31–34 WG) with a median birth weight of 1750 g (IQR: 1360–2050 g). Parents were from a higher socioeconomic level for 28.1% of the children. There were significant differences between children with or without GSA scores for gestational age, social benefits, and level of urbanicity. Table 2 shows descriptive statistics of study variables according to the urbanicity level of the residence.

**Table 1:** Study population characteristics.

Variable	Category	With GSA score n (%)	Without GSA score n (%)	χ2 test (P value)
**Level of urbanicity**	Rural residence	491 (38.2)	79 (27.4)	**< 0.001**
	Quasi-rural residence	329 (25.6)	68 (23.6)	
	Urban residence	466 (36.2)	141 (49)	
**Gender**	Female	611 (47.5)	138 (47.9)	0.953
	Male	675 (52.5)	150 (52.1)	
**Multiple pregnancies**	No	809 (62.9)	171 (59.4)	0.293
	Yes	477 (37.1)	117 (40.6)	
**Gestational age**	22–28 WG	195 (15.2)	39 (13.5)	**0.002**
	29–30 WG	192 (14.9)	64 (22.2)	
	31–32 WG	467 (36.3)	77 (26.7)	
	33–34 WG	432 (33.6)	108 (37.5)	
**Birthweight Z-score**	< −1	306 (23.8)	68 (23.6)	0.55
	−1–0	452 (35.1)	111 (38.5)	
	0–1	416 (32.3)	90 (31.2)	
	> 1	112 (8.7)	19 (6.6)	
**Social benefits for low financial income**	No	1196 (93)	252 (87.5)	**0.003**
	Yes	90 (7)	36 (12.5)	
**Socioeconomic level**	Higher level	924 (71.9)	208 (72.2)	0.957
	Lower level	362 (28.1)	80 (27.8)	

**Table 2:** Study population characteristics according to the urbanicity level of the residence.

		Rural residence (N = 491)	Quasi-rural residence (N = 329)	Urban residence (N = 466)	Total (N = 1286)	
Variable	Category	n (%)	n (%)	n (%)	n (%)	**P.value** $
**GSA score**	Median (IQR)	53 (47,57)	52 (45,56)	53 (45,57)	53 (46,57)	**0.039**
**Gender**	Female	214 (43.6)	178 (54.1)	219 (47)	611 (47.5)	**0.012**
	Male	277 (56.4)	151 (45.9)	247 (53)	675 (52.5)	
**Multiple pregnancies**	No	304 (61.9)	206 (62.6)	299 (64.2)	809 (62.9)	0.765
	Yes	187 (38.1)	123 (37.4)	167 (35.8)	477 (37.1)	
**Gestational age**	22–28 WG	78 (15.9)	52 (15.8)	65 (13.9)	195 (15.2)	0.201
	29–30 WG	75 (15.3)	53 (16.1)	64 (13.7)	192 (14.9)	
	31–32 WG	194 (39.5)	109 (33.1)	164 (35.2)	467 (36.3)	
	33–34 WG	144 (29.3)	115 (35)	173 (37.1)	432 (33.6)	
**Birthweight Z-score**	< −1	92 (18.7)	89 (27.1)	125 (26.8)	306 (23.8)	**0.023**
	−1–0	177 (36)	112 (34)	163 (35)	452 (35.1)	
	0–1	174 (35.4)	107 (32.5)	135 (29)	416 (32.3)	
	> 1	48 (9.8)	21 (6.4)	43 (9.2)	112 (8.7)	
**Social benefits for low financial income**	No	467 (95.1)	309 (93.9)	420 (90.1)	1196 (93)	**0.008**
	Yes	24 (4.9)	20 (6.1)	46 (9.9)	90 (7)	
**Socioeconomic level**	Higher level	388 (79)	243 (73.9)	293 (62.9)	924 (71.9)	**<0.001**
	Lower level	103 (21)	86 (26.1)	173 (37.1)	362 (28.1)	

^$^: Pearson's chi-squared test for categorical variables and Kruskal-Wallis test for quantitative variables

### Urbanicity and the GSA score

**Fig 2:**
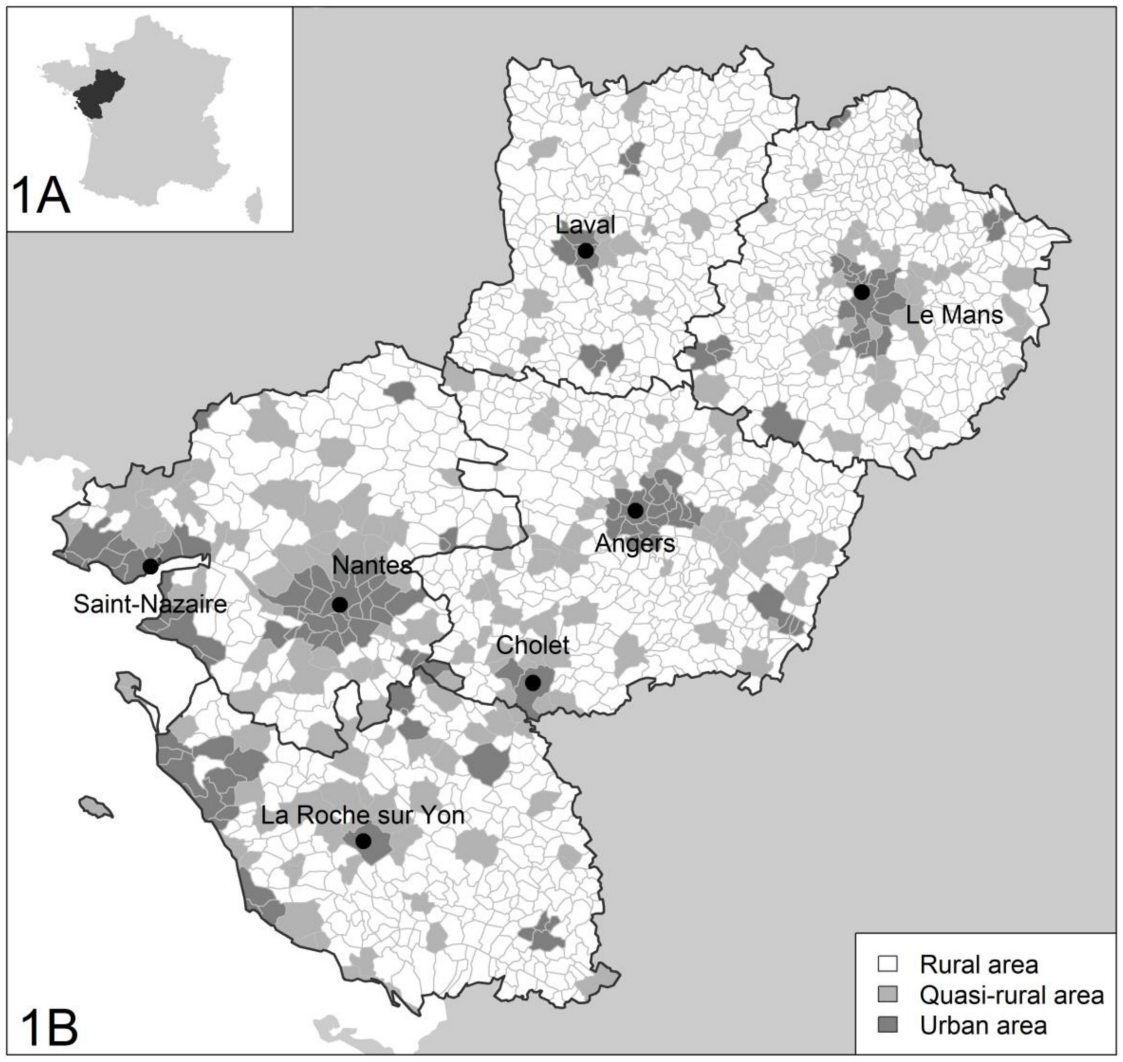
A) Localization of the PDL region in France B) Urbanicity and major cities in the PDL region.

**Fig 3:**
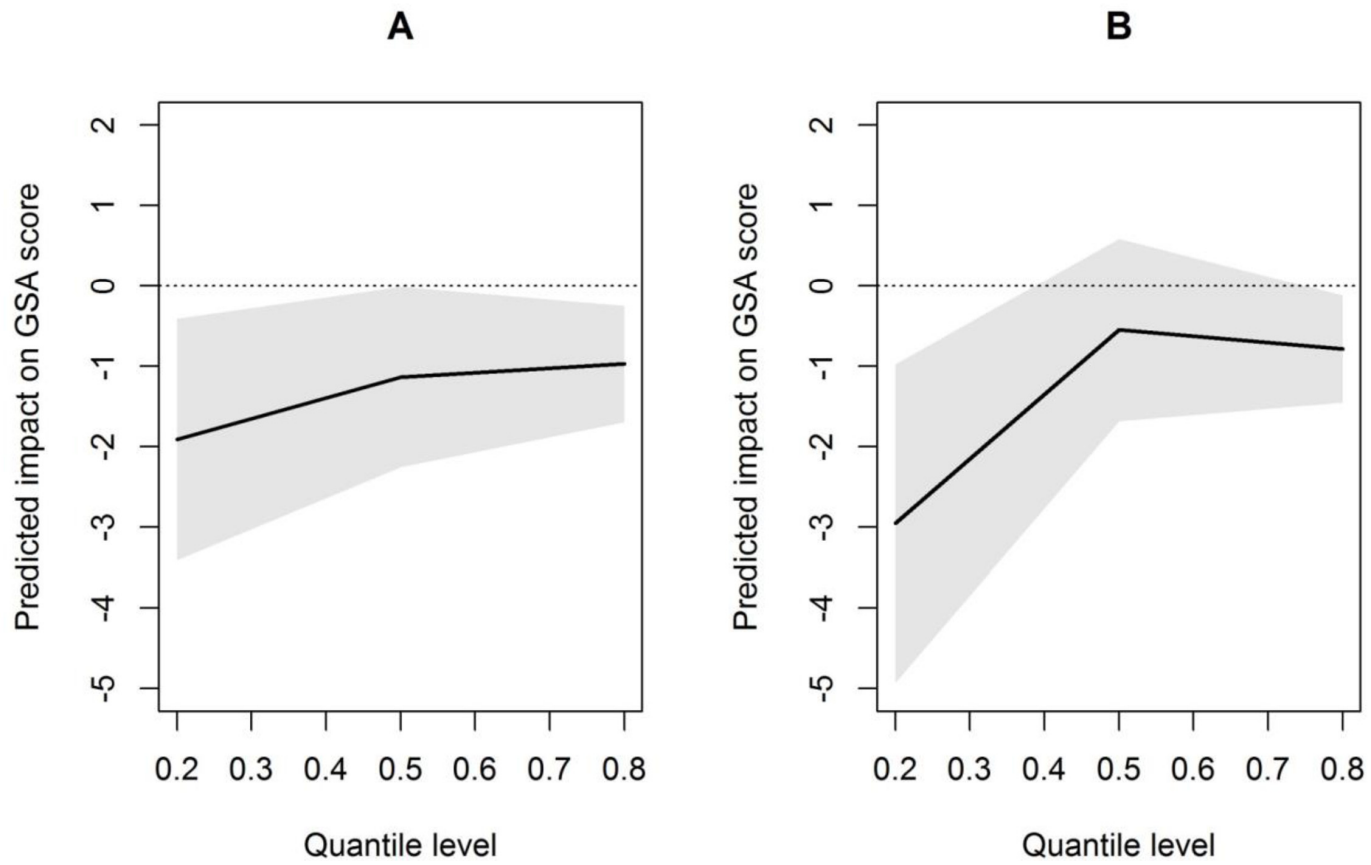
Association between the urbanicity of the residence and the GSA score at age five using model 3. The x-axis represents the quantile level (e.g., 0.2 = 20^th^ percentile) of the GSA score and the y-axis represents the corresponding estimated impact of the level of urbanicity on the GSA score compared to the residence (Panel A: quasi-rural residence, Panel B: urban residence). Panels A-B show the results from a mixed quantile model adjusted for child and parent/family characteristics (model 3). The dotted lines represent pointwise 95 percent confidence intervals.

The urban classifications of communes from the PDL region are reported in Fig 2. Table 3 shows the associations of the residences with the GSA score according to three percentiles (20^th^, 50^th^, and 80^th^) for the three models, which examined the urbanicity level of the residence (model 1), the urbanicity level of the residence adjusted for child characteristics (model 2), and the urbanicity level of the residence adjusted for child and family characteristics (model 3). This table reflects the changes in the GSA score associated with quasi-rural and urban residences compared to rural ones. The urbanicity level of the residence was significantly associated with the GSA score at five years of age in the univariate and multivariate analyses. Using model 3, a residence classified as “urban” had a strong negative association with the GSA scores at five years of age for the lower quantile (20^th^ percentile) and upper quantile (80^th^ percentile), but this association was not significant for the middle quantile (50^th^ percentile). Similarly, a residence classified as “quasi-rural” had a negative association with the GSA scores at age five for all of the percentiles considered (20^th^, 50^th^, or 80^th^ percentile). The negative impact of urbanicity on the GSA score seemed to be more important in the 20^th^ percentile than in the others (Fig 3).

**Table 3:** Association between urbanicity and cognitive development at age five in preterm children born between March 2003 and December 2008 who were free of any disabilities or neurodevelopmental delays at age two using the regional LIFT network. The ß reflect the changes in the GSA score associated with levels of a particular factor compared to predefined reference levels.

		MODEL 1 $						MODEL 2 £						MODEL 3 ¤					
		20^th^ percentile		50^th^ percentile		80^th^ percentile		20^th^ percentile		50^th^ percentile		80^th^ percentile		20^th^ percentile		50^th^ percentile		80^th^ percentile	
Variable	Category	ß	SE	ß	SE	ß	SE	ß	SE	ß	SE	ß	SE	ß	SE	ß	SE	ß	SE
**Urbanicity**	Rural residence	0	-	0	-	0	-	0	-	0	-	0	-	0	-	0	-	0	-
	Quasi-rural residence	−**1.92** *	0.83	−0.77	0.63	−0.78*	0.45	−**2.07** *	0.80	−**1.20** *	0.56	−**0.97** *	0.36	−**1.91** *	**0.76**	−**1.14** *	0.57	−**0.97** *	0.37
	Urban residence	−**2.92** *	1.17	−0.77	0.65	−0.79*	0.45	−**2.53** *	1.27	−0.27	0.55	−**0.79** *	0.35	−**2.95** *	1.01	−0.55	0.58	−**0.79** *	0.34
**Child sex**	Female	-	-	-	-	-	-	0	-	0	-	0	-	0	-	0	-	0	-
	Male	-	-	-	-	-	-	−5.46*	0.67	−4.30*	0.47	−1.60*	0.33	−5.02*	**0.64**	−**4.21**	0.49	−1.73*	0.32
**Gestational Age #**	≤ 28	-	-	-	-	-	-	−3.00*	0.97	−2.07*	0.76	−1.44*	0.56	−3.09*	**0.91**	−1.84*	0.80	−1.65*	0.60
	29–30	-	-	-	-	-	-	−0.53	0.95	−0.57	0.69	−0.83	0.48	−0.98	0.99	−0.61	0.73	−0.71	0.49
	31–32	-	-	-	-	-	-	0.88	0.88	0.72	0.65	−0.04	0.40	0.91	0.85	0.65	0.63	0.03	0.41
	33–34	-	-	-	-	-	-	0	-	0	-	0	-	0	-	0	-	0	-
**Birthweight Z-score**	< −1	-	-	-	-	-	-	−2.76*	0.94	−2.15*	0.83	−1.32*	0.60	−3.11*	**0.94**	−2.01*	0.82	−1.16*	0.61
	[−1, 0]	-	-	-	-	-	-	−2.77*	0.80	−1.54*	0.72	−0.54	0.57	−3.09*	**0.8**	−1.62*	0.71	−0.38	0.55
	[0, 1]	-	-	-	-	-	-	−2.25*	0.90	−0.82	0.68	−0.36	0.53	−2.13*	0.87	−0.8	0.70	−0.38	0.52
	> 1	-	-	-	-	-	-	0	-	0	-	0	-	0	-	0	-	0	-
**Multiple pregnancies**	No	-	-	-	-	-	-	0	-	0	-	0	-	0	-	0	-	0	-
	Yes	-	-	-	-	-	-	0.59	0.67	0.09	0.51	−0.18	0.33	0.01	0.7	0.15	0.50	−0.21	0.31
**Social benefits for low financial income**	No	-	-	-	-	-	-	-	-	-	-	-	-	0	-	0	-	0	-
	Yes	-	-	-	-	-	-	-	-	-	-	-	-	−1.47	1	−1.13	0.94	−0.76	0.74
**Socio-economic level**	Higher level	-	-	-	-	-	-	-	-	-	-	-	-	0	-	0	-	0	-
	Lower level	-	-	-	-	-	-	-	-	-	-	-	-	2.02*	0.72	1.81*	0.50	0.46	0.33

* p < 0.05;

^**$**^ **Model 1**: Urbanicity variable;

^**£**^ **Model 2**: Urbanicity and child variables;

^**¤**^ **Model 3**: Urbanicity, child, and parent/family variables; **SE** = standard error; **#** Weeks of gestation

The lower quantile of the GSA score was also associated with male gender, low gestational age, low Z-score birthweight, and low socioeconomic level (Table 3).

### Contextual factors of urbanicity

A high socioeconomic deprivation level of the residential area was significantly associated with the GSA score at five years of age only for the lower quantile (20^th^ percentile) (Table 4). Access to both GP and MCH clinics were not associated with the GSA score at five years of age, regardless of the studied percentile.

**Table 4:** Association between the contextual effects of urbanicity and cognitive development at age five in preterm children born between March 2003 and December 2008 who were free of any disabilities or neurodevelopmental delays at age two using the regional LIFT network. The ß reflect the changes in the GSA score associated with levels of a particular factor compared to predefined reference levels.

		MODEL 4: Deprivation index $						MODEL 5: GP index $						MODEL 6: Distance to MCH clinic $					
		20^th^ percentile		50^th^ percentile		80^th^ percentile		20^th^ percentile		50^th^ percentile		80^th^ percentile		20^th^ percentile		50^th^ percentile		80^th^ percentile	
Variable	Category	ß	SE	ß	SE	ß	SE	ß	SE	ß	SE	ß	SE	ß	SE	ß	SE	ß	SE
**Deprivation**	Low	0	-	0	-	0	-	-	-	-	-	-	-	-	-	-	-	-	-
	Medium	-0.95	0.84	-0.49	0.61	-0.73	0.45	-	-	-	-	-	-	-	-	-	-	-	-
	High	-2.46*	1.13	-0.81	0.74	-0.74	0.50	-	-	-	-	-	-	-	-	-	-	-	-
**GP accessibility**	Low	-	-	-	-	-	-	0.39	0.90	0.39	0.63	0.85	0.49	-	-	-	-	-	-
	Medium	-	-	-	-	-	-	0.96	1.08	0.54	0.58	0.46	0.41	-	-	-	-	-	-
	High	-	-	-	-	-	-	0	-	0	-	0	-	-	-	-	-	-	-
**Distance to MCH clinic**	Low	-	-	-	-	-	-	-	-	-	-	-	-	0	-	0	-	0	-
	Medium	-	-	-	-	-	-	-	-	-	-	-	-	-0.67	1.03	0.02	0.61	0.54	0.39
	High	-	-	-	-	-	-	-	-	-	-	-	-	-0.72	1.04	0.03	0.73	0.62	0.44

* p < 0.05;

^**$**^ models adjusted for child sex, gestational age, birthweight Z-score, multiple pregnancies, social benefits for low financial income, and socioeconomic level

## Discussion

In this large population-based cohort of children born before 35 weeks of gestation, the level of urbanicity was associated with disparities in cognitive development at five years of age in univariate and multivariate analyses. Indeed, living in urban or quasi-rural areas was associated with a lower GSA score compared to living in rural ones. To examine this link, we used novel quantile regression methods. The retrieved parameter estimates differed across the percentiles, suggesting that quantile regression methods were appropriate to describe the associations between all of the studied explanatory variables and the GSA score. The parameter estimates were fairly stable across the different computed models for urbanicity, suggesting that there was little effect of the child and family characteristics on the association of urbanicity with cognitive development. Its impact was more prominent in the 20^th^ percentile of the GSA score. Indeed, in this quantile, living in urban areas was associated with a three point diminution of the GSA score. This emphasizes the large potential impact of urbanicity on the most vulnerable preterm children in our study population (those with the lowest GSA scores). Because the median GSA score was around 30 for these children, we roughly estimate that living in urban areas compared to rural ones is associated with a 10% diminution of the GSA score, after adjusting for children and parent/family characteristics.

The main limitation of this study was the number of children without valid GSA data (288 children) and the significant differences in their characteristics compared to the children with valid GSA data. For children for whom parents were from a lower socioeconomic level or with a gestational age of 33–34 WG, those that lived in an urban area were more represented in the group without a GSA score. However, because our study was a population-based study of 1286 children with a good distribution of children in the three urbanicity groups, this bias was limited.

A second limitation could be the use of the GSA score to assess neurodevelopment at five years of age. First, the GSA score may be influenced by subjective factors such as the relationship between the child and their teacher. Indeed, for the GSA score, the assessor is the teacher who has known and seen the child every day. This score thus also partly reflect the expectations of the teacher in addition to the true cognitive development of the child. Second, parents have to give the questionnaire to the teacher and then return it to the hospital; this can explain the number of children without a GSA score at age five. Third, there could also be a positivity bias, which is the tendency to reduce a negative judgment to make it more socially acceptable for oneself and for others. This could affect the GSA evaluation made by the teachers [32]. This type of bias has previously been shown in another French study that used a similar evaluation for severe problems [33]. Nevertheless, the GSA score is a simple screening tool for preterm children. This score assesses the child in their own environment and compares the child with other children from the same school class who constitute the control group. It is of particular interest for the behavior and socialization of the children [25]. This characteristic limits the possibility of a differential rating between children from rural, semi-rural and urban place of residences. Furthermore, the previously described positivity bias indirectly reinforces our results. Indeed, this bias would only underestimate the impact of urbanicity on cognitive development. In this study, a lower GSA score was associated with low gestational age and Z-score birthweight, male gender, and a low socioeconomic level. These findings are in agreement with previous studies from the literature [34].

A third limitation is that the chosen scale of analysis cannot reveal large disparities in environmental influences. This is particularly true in urban areas. To clearly identify the contextual factors affecting the cognitive development of preterm children, studies that take into account the intra-urban disparities in the social, physical, and health accesses must be performed. Nevertheless, our chosen scale of analysis provides a simple and reliable prevention message to the medical community for preterm children.

A fourth limitation is that only the most recent place of residence is available for each child included in our cohort. Consecutively, a quantification of the bias induced by potential changes of residence during the first 5 years of life is not possible.

In this study, we chose to study a population of preterm children free of any disabilities or neurodevelopmental delays because this population is not well studied and is at risk of mild delays in multiple areas of development and cognitive impairment, which can lead to school difficulties [12–14]. The absence of any disabilities and neurodevelopmental delays at age two allowed us to overcome the perinatal risk factors. Moreover, we suggest that this vulnerable population is more sensitive to their environmental context. To the best of our knowledge, this is the first study to identify the impact of contextual factors on the cognitive development in this population.

Urbanicity has both adverse and beneficial effects on health: the beneficial effects include better access to health care and educational and social support; the adverse ones include poor physical activity and dietary habits and high exposure to pollution, noise, and anxiety [35]. In this study, we also tried to identify the impact of specific contextual characteristics of urbanicity on the cognitive development of preterm infants. The most common topics of concern that emerge from the urban health literature are separated in three principal themes: features of the social environment, features of the physical environment, and the provision of health and social services [20]. In our study, even after controlling for relevant personal and family background characteristics, a highly deprived place of residence was shown to have negative effects on developmental outcomes of preterm children.

The non significance of this relation when controlling for the urban level of residence reinforces the hypothesis that a large part of the link between urbanicity and cognitive development is mediated by the social environment (Annex 2). This result is in accordance with previous studies performed on children from the general population [36]. At the contrary, both access to GP and MCH clinics (reflecting the provision of health services) were not associated with the GSA score at five years of age in preterm children. Although more in-depth investigations are needed, these negative results seem to suggest that the provision of medical services is not a major explanation for the observed difference in cognitive development between rural and urban place of residences.

The composite socioeconomic deprivation index used in our analysis is nevertheless only an incomplete reflection of the children social environment. Others individual or group social characteristics not taken into account in our analysis (such as ethnicity, number of brothers and sisters, number of divorces, degree of physical activity or sports participation) may also explained in a significant part the observed results. Further studies are clearly needed to quantify more precisely the impact of social environment on the cognitive development of preterm children.

Due to a high correlation with the social environment, a significant impact of the physical environment as well as of the provision of social services (such as education) on the cognitive development of preterm children could nevertheless not be excluded.

Indeed, a growing number of studies have documented the significant effects of the physical environment on the health and outcomes in children [16–18]. In urban areas, exposure to high levels of pollution were associated with lower IQ scores at age five [37,38]. In 2006, Evans et al. demonstrated that chronic noise exposure in urban areas interfered with reading acquisition and impacted the persistence and effort needed for academic achievement in children. These authors also showed that a crowded home negatively impacted the behavior of children and decreased their academic achievement [39]. Data characterizing the physical environment at the commune level of the children were not available, preventing investigation of its impact on the cognitive development of preterm children in our study. Future studies are needed to investigate this link.

Additionally, study results may alternatively be explained by the differential quality of the school education between the rural and urban areas. Because the population in urban areas is growing, schools in French urban areas have to tackle the problem of overcrowded classes and oversized schools. It was shown that reduced class sizes facilitate individualized instruction, higher quality instruction, greater scope for innovation and child-centered teaching, increased teacher morale, fewer disruptions, less misbehavior, and greater ease in engaging children in academic activities [40]. By contrast, schools in French rural areas suffer from a shortage of students because of the population decline. Since a teacher can spend more time with each child, smaller school sizes would be a benefit for children [41]. These associated consequences of oversized classes may be more important for children with cognitive difficulties who need more attention. However, further studies incorporating school-level data are necessary to confirm this alternative hypothesis.

## Conclusion

In summary, we conducted multi-level quantile regression analyses to assess the association between urbanicity and cognitive development in five-year-old preterm children born before 35 WG who were free of any disabilities or neurodevelopmental delays at age two. Using this novel method, we found that living in urban and quasi-rural areas was associated with lower cognitive development, with the strongest association at the lower tail of the GSA score distribution. This is an important finding that suggests that the environment has an important impact on the cognitive outcome in preterm children born before 35 WG. This also suggests, if these results are confirmed, that a more personalized follow-up could be developed, particularly in children with a low GSA score at five years of age who live in highly deprived urban or quasi-rural areas. The strong influence of social factors on cognitive function suggests that gains in cognitive development depend mainly on efforts to redress disadvantages in the child’s social environment. However, more studies are needed to clearly identify the contextual characteristics of urbanicity that explain this association.
